# Transcriptomic and in vivo approaches introduced human iPSC-derived microvesicles for skin rejuvenation

**DOI:** 10.1038/s41598-023-36162-9

**Published:** 2023-06-20

**Authors:** Behnaz Bakhshandeh, Zohreh Jahanafrooz, Shiva Allahdadi, Shiva Daryani, Zahra Dehghani, Mahya Sadeghi, Mir Sepehr Pedram, Mohammad Mehdi Dehghan

**Affiliations:** 1 Department of Biotechnology, College of Science, University of Tehran, P.O. Box 14155-6455, Tehran, Iran; 2 Department of Biology, Faculty of Sciences, University of Maragheh, Maragheh, Iran; 3 Department of Cellular and Molecular Biology, School of Biology, College of Science, University of Tehran, Tehran, Iran; 4 Department of Biomedical Engineering, Central Tehran Branch, Islamic Azad University, Tehran, Iran; 5 Department of Surgery and Radiology, Faculty of Veterinary Medicine, University of Tehran, Tehran, Iran; 6 Institute of Biomedical Research, University of Tehran, Tehran, Iran

**Keywords:** Biological techniques, Biotechnology, Cell biology, Drug discovery, Genetics, Molecular biology, Stem cells

## Abstract

The skin undergoes the formation of fine lines and wrinkles through the aging process; also, burns, trauma, and other similar circumstances give rise to various forms of skin ulcers. Induced pluripotent stem cells (iPSCs) have become promising candidates for skin healing and rejuvenation due to not stimulating inflammatory responses, low probability of immune rejection, high metabolic activity, good large-scale production capacity and potentials for personalized medicine. iPSCs can secrete microvesicles (MVs) containing RNA and proteins responsible for the normal repairing process of the skin. This study aimed to evaluate the possibility, safety and effectiveness of applying iPSCs-derived MVs for skin tissue engineering and rejuvenation applications. The possibility was assessed using the evaluation of the mRNA content of iPSC-derived MVs and the behavior of fibroblasts after MV treatment. Investigating the effect of microvesicle on stemness potential of mesenchymal stem cells was performed for safety concerns. In vivo evaluation of MVs was done in order to investigate related immune response, re-epithelialization and blood vessel formation to measure effectiveness. Shedding MVs were round in shape distributed in the range from 100 to 1000 nm in diameter and positive for AQP3, COL2A, FGF2, ITGB, and SEPTIN4 mRNAs. After treating dermal fibroblasts with iPSC-derived MVs, the expressions of collagens Iα1 and III transcripts (as the main fibrous extracellular matrix (ECM) proteins) were upregulated. Meanwhile, the survival and proliferation of MV treated fibroblasts did not change significantly. Evaluation of stemness markers in MV treated MSCs showed negligible alteration. In line with in vitro results, histomorphometry and histopathology findings also confirmed the helpful effect of MVs in skin regeneration in the rat burn wound models. Conducting more investigations on hiPSCs-derived MVs may lead to produce more efficient and safer biopharmaceutics for skin regeneration in the pharmaceutical market.

## Introduction

The skin serves as a barrier against infection and exogenous chemicals and physical invasions, so skin injuries need to be repaired immediately and appropriately to maintain its protective roles. Epidermis, dermis, and subcutaneous tissue are the three main layers of the skin. Epidermis is the outer section of the skin that mainly consists of multiple layers of keratinocytes. Skin stem cells compromise the deepest layer of the epidermis which forms all cell types in the epidermis^1^. Dermis is a complex layer involving hair follicles, nerves, blood vessels, sweat and sebaceous glands, muscles, fibroblast, and ECM. Dermal fibroblasts have a substantial role in constructing the ECM of the skin, which contains two major classes of biomolecules; glycosaminoglycans that form proteoglycans by binding to proteins mainly through covalent bonds, and fibrous proteins, like fibronectin, elastin, laminin, and collagen^2^.

Different circumstances, such as trauma, burns, and certain diseases, can cause various forms of skin ulcers^1^. In addition, the aging process results in thinning of dermis and epidermis. The underlying fat layer can be lost as well. The decrease in volume and overall quality of all three skin layers results in a number of changes in the color and texture of the skin. It becomes drier due to impaired barrier function and decreased production of essential oil like sebum. The number of sweet glands and blood vessels decreases as well, reducing the skin’s ability to respond to heat exposure. All of these changes make the skin more susceptible to damage and slower to heal; hence, skin rejuvenation methods and safe approaches for the treatment of skin wrinkles and wounds are currently in the spotlight^3–6^.

Wound-healing is defined as a process associated with proliferation, inflammation, and remodeling; furthermore, a normal wound healing process in skin involves complex interactions between cellular (such as skin stem cells and fibroblasts which contribute to reduce the contraction of the wound and support collagen synthesis and neovascularization^7^) and acellular (such as growth factors and cytokines) components. The coordinated interactions within the skin enable healing process to happen naturally or as a result of various therapies. Skin repair mechanisms and related signaling pathways are mediated by different genes, such as AQP3 (encoding a protein responsible for transporting water and glycerol, participating in the skin lipid metabolism, and regulating the differentiation and proliferation of keratinocytes in the wound-healing process), COL2A (encoding the main fibrous protein of skin ECM), FGF2 (encoding an angiogenic and mitogenic factor stimulating the cells proliferation and migration at the wound site), ITGB (encoding the main ECM adhesion transmembrane molecule), SEPTIN4 (encoding a GTP-binding protein involved in the morphogenesis and physiological functions of many cells, like skin cells), as well as FGF7, FGFR2, PPARD, and STAT3^8–16^. Any approach that provides the mentioned transcripts or induces their expression in target cells can help skin rejuvenation and wound healing.

Stem cell-based therapy can be a potent option for wound-healing and skin rejuvenation^17–19^. Induced pluripotent stem cells (iPSCs) can directly be produced from mature cells, propagate independently, and give rise to other kinds of cells; moreover, due to a lack of documented evidence for graft rejection and inflammatory responses, they hold considerable promise in personalized regenerative medicine^20–22^. In this regard, some important skin cells, like keratinocytes, fibroblasts, and melanocytes, have been produced from iPSCs^23–25^.

The release of vesicles, including microvesicles (MVs), exosomes, and apoptotic bodies, is a conserved process in almost all cell types^26–28^. MVs are practical communication tools that participate in cell interactions and influence the performance and physiological features of the recipient cells; they also contain different amounts of genetic products, proteins, and other substances^28–30^. Studies have demonstrated that MVs have pivotal roles in the remodeling of ECM in wound-healing through their involvement in coagulation, cell proliferation, and cell migration processes^31–33^. The high metabolic activity of iPSCs and subsequent enormous shed-MVs, and large-scale production capacity along with their merits in personalized medicine, have prompted us to utilize iPSCs-derived MVs for skin rejuvenation and regeneration approaches.

**Figure 1:**
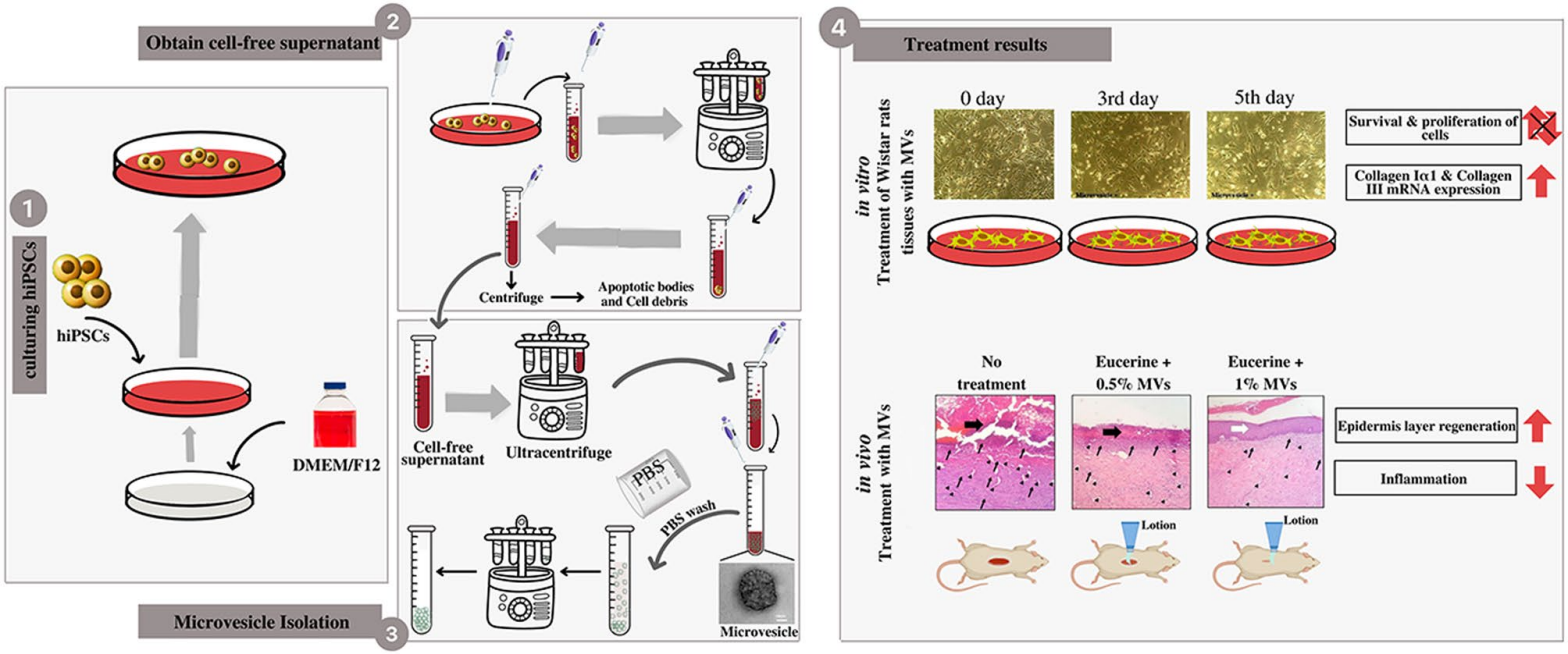
Overview of the procedure of isolating MVs from human iPSCs and the effects of hiPSCs-derived MVs both in vitro and in vivo. After treating dermal fibroblasts with MVs, the in vitro analysis showed that the rate of collagen expression had been increased, and the survival and proliferation alterations were not considerable. Within in vivo trials, the wounds created on the dorsal skin of the rats were treated by lotions containing Eucerin and different amounts of MVs. The in vivo analysis showed an increased rate of epidermis layer regeneration and decreased rate of inflammation in the wound site of mice treated with higher amounts of MVs. MVs, microvesicles; iPSCs, induced pluripotent stem cells; hiPSCs, human induced pluripotent stem cells; ECM, extracellular matrix.

Herein we evaluated the possibility, safety and effectiveness of applying iPSCs-derived MVs for skin tissue engineering and rejuvenation applications. The possibility was assessed using the evaluation of the mRNA content of iPSC-derived MVs and the behavior of fibroblasts after MV treatment. Investigating the effect of microvesicle on stemness potential of mesenchymal stem cells was performed for safety concerns. In vivo evaluation of MVs was done in order to investigate related immune response, re-epithelialization and blood vessel formation to measure effectiveness (Fig. 1).

## Materials and methods

### Cell culture and characterization

Human iPSCs (gifted from Bonyakhteh Stem Cell Research Center, Tehran, Iran) were maintained on mitomycin C-arrested mouse embryonic fibroblasts in an undifferentiated state^21^ in DMEM/F12 (Gibco by Life Technologies™, Paisley, Scotland, UK) medium supplemented with 1% non-essential amino acids, 20% knockout serum replacement, 4 ng/ml bFGF (all from Sigma-Aldrich Chemie GmbH, Steinheim, Germany), and 1 mM L-glutamine (Sigma-Aldrich, St. Louis, USA). MSCs isolation from young rat bones, cell surface marker evaluation, and investigation of their multi-linage differentiation capacity were performed as previously reported^34–37^. DMEM/F12 medium (supplemented with fetal bovine serum (20%) and penicillin/streptomycin (1% v/v) (both from Gibco by Life Technologies™, Paisley, Scotland, UK) was utilized for culturing isolated MSCs. MSCs were seeded in a 24-well plate with 35 × 10^3^ cells/well density after the second passage . Human fibroblasts (purchased from Bonyakhteh Stem Cell Research Center, Tehran, Iran) were cultured according to a similar protocol for the MSC culture.

### Microvesicle isolation

Cell cultures were replaced with the serum-free medium about 8 h before centrifugation for minimizing the FBS albumin contamination. Then, the cell-free supernatant of the hiPSCs culture was obtained after 2000 rpm centrifugation at 4 °C for 10 min. Apoptotic bodies and cell debris were removed by a 10,000 *g* centrifugation at 4 °C for 20 min. MVs pellets were obtained after 60,000 *g* centrifugation at 4 °C for one hour and were PBS (phosphate-buffered saline)-washed, then centrifugation was repeated at 60,000 g. It is noticeable that fresh pellets were used for each treatment.

To estimate the approximate concentration of MVs in the pellets, we measured the protein content of the pellets as an indirect equalizer using the Bradford assay. For each treatment, 20 μg/ml (protein content/medium volume) pellets were added to each well of the cultured cells.

### Transmission electron microscopy (TEM)

Both size and morphology of MVs were characterized via TEM. For this purpose, a drop of the suspended MV pellet was stained with 2% -uranyl acetate on formvar-carbon coated grids. After drying, transmission images were provided by placing the grid in an electron microscope (Philips cm30).

### Dynamic light scattering (DLS)

The DLS was performed to analyze the size range and homogeneity of the isolated MV population using the Malvern instrument.

### RNA content of the isolated MVs

The RNA content of the MVs was extracted using the RNX Plus™ kit (SinaClon, Tehran, Iran). Due to low amounts of extracted mRNAs, the detection of specific mRNA content of MVs was performed according to SinaClon First Strand cDNA Synthesis Kit (SinaClon, Tehran, Iran) and qPCR Master Mix (A320799 Ampliqon, Denmark). Designed primers for specific mRNAs are depicted in supplementary Table 1. Quality control of the primers was carried out by LinReg PCR software (Amsterdam, Netherlands).

### MVs treatment of the cells

To investigate the effect of MVs derived from hiPSCs supernatant on MSCs and fibroblasts, roughly 35 × 10^3^ cells/well got seeded in 24-well plates, and the tests were conducted on days 1, 3, and 5 and repeated three times for each. 20 μg/ml (protein content/medium volume) of pellets were supplemented to cultured cells, for each treatment.

### Cell proliferation and viability assessments

The MTT (Sigma-Aldrich, St. Louis, USA) test was conducted to assess the possible toxic effects of MVs on fibroblasts metabolic activity. Fibroblasts were seeded at a density of 35 × 10^3^ cells/well in a 24-well plate. On days 1, 3, and 5 after incubation, 30 µl of MTT solution (5 µg/ml) was added to cell plate wells and incubated for 4 h at 37 °C. Afterward, 100 µl DMSO (solubilization buffer) (Sigma-Aldrich, St. Louis, USA) was added at room temperature and shaken for 15 min. Thereafter, the absorption was measured with a spectrophotometer at 570 nm. The darker the solution, the greater the number of viable, metabolically active cells^38^. The trypan blue test was also applied to figure out if MVs treatment had any impacts on fibroblast viability. The attached cells were washed using PBS (pH 7.4), then trypsinized with TrypLE™ (GIBCO Corp) for 1 min at 37 °C; finally, FBS-supplemented culture media was utilized for their neutralization. For the determination of viable cells, the cells were stained with 4% Trypan blue and counted manually by a hemocytometer.

### Evaluation of cell apoptosis by Annexin V assay

To investigate the possible role of MVs treatment on apoptosis induction in fibroblasts, on days 1, 3, and 5 of culture, Annexin V-FITC and PI (EXBIO, Vestec, Czech Republic) were utilized for double-staining of cells, followed by flow cytometry (BD Biosciences, San Jose, CA, USA) analysis.

### Evaluation of gene expression changes in MV-treated cells

Synthesis of cDNA and isolation of total RNA was carried out using the kits (SinaClon, Tehran, Iran), based on instructions prepared by the manufacturer. The qPCR reactions were conducted utilizing RealQ PCR Master Mix (A320799 Ampliqon, Denmark) in Rotor-Gene 6000 Real-Time Thermal Cycler (Corbett Research, Australia). The primers for HPRT, NANOG, collagen I, and collagen III were purchased from the Stem Cell Technology Research Center, Iran. LinReg PCR application (Amsterdam, Netherlands) was utilized for primer quality evaluation. Relative Expression Software Tool (REST 2009, Corbett Research, Australia) was used to measure relative gene expression through the 2^-∆∆Ct^ approach.

### In vivo assays

60 adult male rats with an average weight of 300–350 g and an average age of 3–4 months were divided were used in this experiment. All the animals were kept for one week under stable environmental (25 °C temperature, 50% humidity) and nutritional conditions with free access to food and water. After habituation, animals were randomly divided into four groups as described in Table 1. In all rats, the skin of the dorsum was shaved and a standard deep 2nd degree burn wound was made using a hot aluminum plate at 95 °C for 6 s. The animals were caged separately and maintained in standard conditions at 20 °C under 12 h periods of darkness/light. Rats were deeply anesthetized with Ketamine (70 mg/ml) and Xylazine (10 mg/ml). All groups were evaluated for 18 days although in each group, some rats died before analyses, particularly because of infection. Animal working procedures were performed at the Faculty of Veterinary Medicine, University of Tehran. All experimental protocols were performed under ethics approval obtained by the Iran Animal Care Committee. Also, experiments were done in accordance with the approved protocols, institutional guidelines for the care and use of laboratory animals and ARRIVE recommendations. Digital imaging was used to determine the appearance of the wound size. The AxioVision Rel. 4.8 software program was used to evaluate the images acquired from the injury sites geometrically.

**Table 1:** description of the analyzed in vivo groups (rats with burn wounds).

Group name	MVs treatment	Eucerin treatment
A	None	None
B	None	0.192 g Eucerin
C	0.5% w/w	0.192 g Eucerin
D	1% w/w	0.192 g Eucerin

### Lotion formulation and preparation

Each rat was treated with 0.192 g Eucerin (Boote Sabze Mehr Aein Company, Iran) and the required amount of MVs according to the related group. For better homogenization of the MVs solution into Eucerin, the MVs solution was freeze-dried. Finally, 0.5 g MV powder was used in all treatments.

### Histopathology analysis

On each timepoint of 1, 4, 8, 12, 14, and 18 days, three rats of each group were sacrificed. The tissue sample of wounds was removed completely and fixed in 10% formalin buffer (pH = 7.26) for 48 h. The samples were embedded into paraffin, and sections were prepared with a thickness of 5 mm. The samples were then stained with hematoxylin and eosin (H&E) with subsequent evaluation by light microscopy (Olympus BX51; Olympus, Tokyo, Japan). Inflammatory cell infiltration, epithelialization, and granulation tissue formation were compared by examining the histopathological slides of all experimental groups.

### Histomorphometry analysis

A semi-quantitative evaluation of epithelialization was reported as a 5-point scale for 18 days: 0 (without new epithelialization), 1 (25%), 2 (50%), 3 (75%), and 4 (100%). One independent observer blinded to the treatment groups approved the parameters comparatively. Furthermore, histomorphometry analysis and neovascularization were calculated and analyzed, using computer software Image-Pro Plus® V.6 (Media Cybernetics, Inc., Silver Spring, USA). Cells were counted at 400X magnification, and the calculation was repeated for four fields to obtain the numerical average of each criterion.

### Statistical analysis

All tests were performed at least in triplicate and the outcomes were presented as mean ± SD. To compare the data of the two or more groups, the two-tailed t-test of students and one-way variance analysis (ANOVA) were applied. Microsoft Excel 2016 software, one statistical online software (http://vassarstats.net/), and GraphPad Prism version 6.01 were used to conduct the statistical analyses and graphical representations. A *p*-value of < 0.05 was considered statistically significant.

### Ethical approval

All experimental protocols were performed under ethics approval obtained by the Iran Animal Care Committee. Also, experiments were done in accordance with the approved protocols, institutional guidelines for the care and use of laboratory animals and ARRIVE recommendations.

## Results

### Characterization of iPSC-Derived MVs

**Figure 2:**
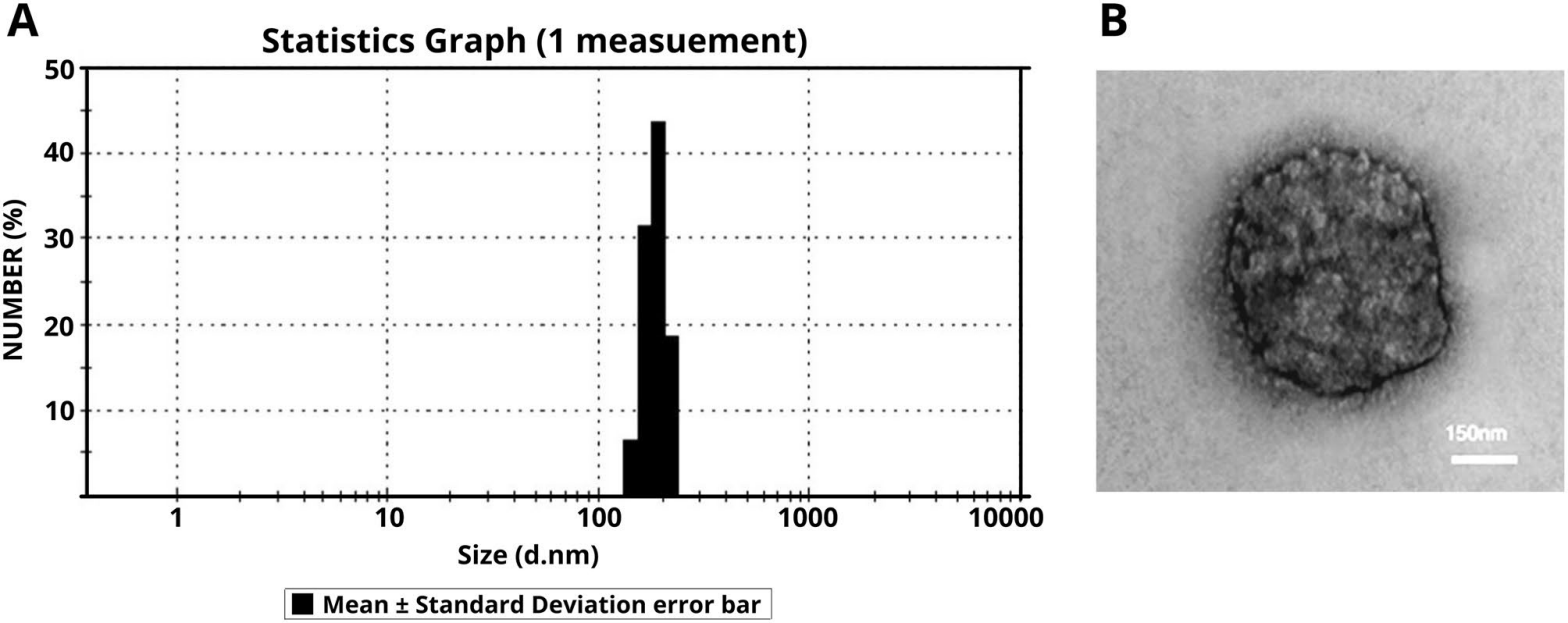
Microvesicle characterization; (**A**) The population homogeneity and size range of isolated MVs obtained by DLS. (**B**) TEM image of a single isolated MV demonstrated a rounded shape.

According to representative micrographs obtained from transmission electron microscopy (Fig. 2), the size of isolated MVs was smaller than 1 μm, indicating the accuracy of the isolation protocol. Additionally, electron microscopy demonstrated the rounded shape of purified MVs. The DLS analysis showed the rages of isolated MVs from 150 to 250 nm with a narrow and sharp peak.

After mRNA isolation from isolated MVs and subsequent cDNA synthesis, nine skin rejuvenation-related genes including AQP3, Col2A, FGF2, FGF7, FGFR2, ITGB1, PPARD, SEPTIN4, and STAT3 were investigated by qPCR. AQP3, COL2A, FGF2, ITGB, and SEPTIN4 transcripts were detected in the graphs (not shown), implying the transferring of these mRNAs by MVs among the cells.

### The effect of MVs on fibroblasts

**Figure 3:**
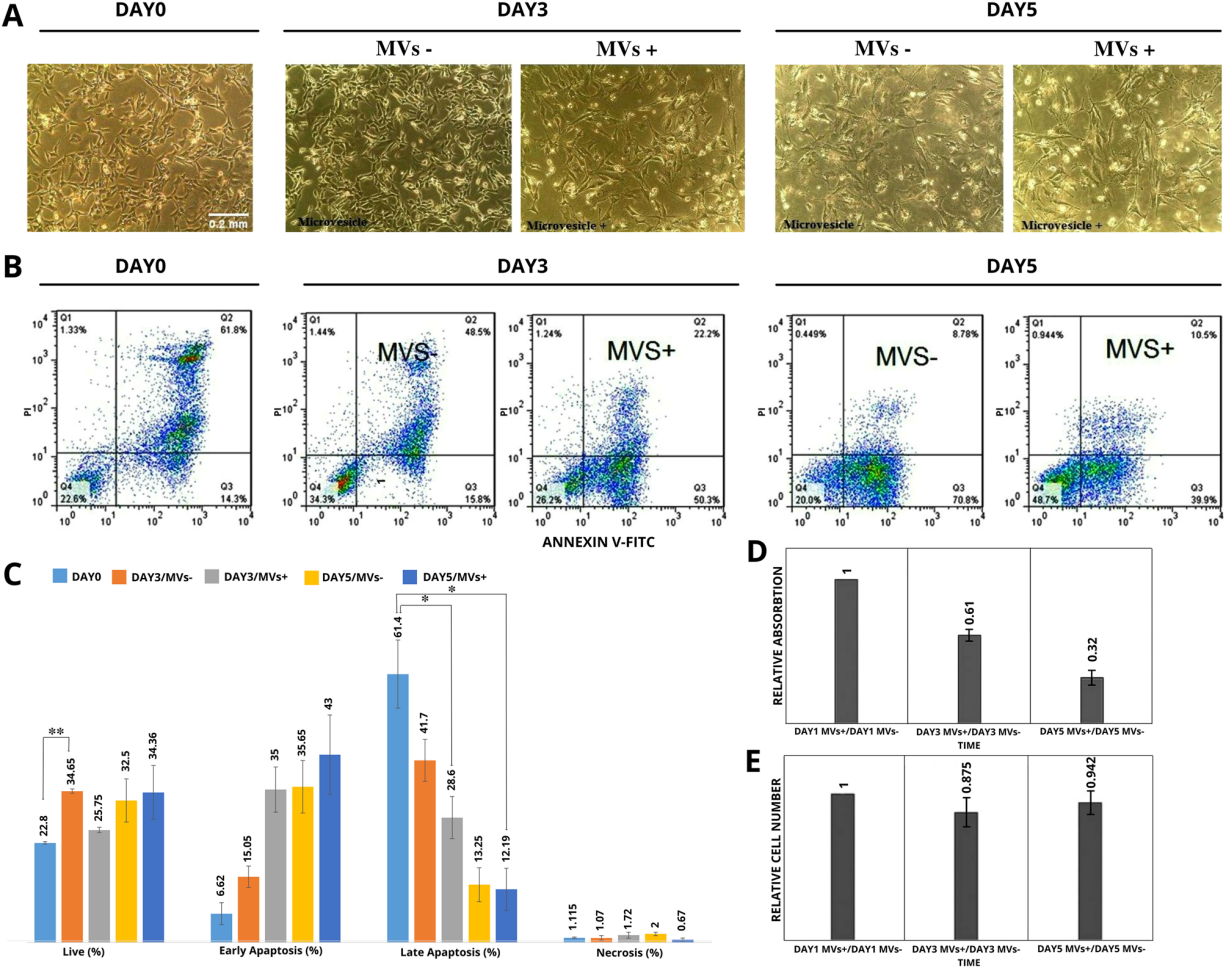
Evaluation of the apoptosis and necrosis of skin fibroblasts under MVs treatment. (**A**) representative images of cultivated MV + and MV- fibroblasts in DMEM/F12 medium on days 0, 3, and 5, scale bars 100 µm (**B**) The rate of apoptosis and necrosis in fibroblasts characterized by Annexin V/PI dual staining flow cytometry on days 0, 3, and 5 and MVs treated groups. For further evaluation, a scattered plot chart is drawn. Q1: necrotic cells (Annexin V-/PI +), Q2: late apoptotic cells (Annexin V + /PI +), Q3: early apoptotic cells (Annexin V + /PI-), Q4: viable cells (Annexin V-/PI-). (**C**) The percentage of viable, early apoptotic, late apoptosis, and necrotic cells on days 0, 3, and 5 and MVs treated groups using a bar chart. Data are statistically significant in the live group on day 0 and day3/MVs- (**P-value < 0.01) and also in late apoptotic MVs treated groups on day 3 and day 5 compared to day 0. (**D**) On days 3 and 5 after MVs treatment cell absorbance at 570 nm had no significant differences. (**E**) Investigation of survival of MVs + and MVs- fibroblasts by Trypan blue assay on days 0, 3, and 5. MVs treatment did not affect fibroblast viability significantly. Data are presented as means ± SD of at least three independent experiments. Data presents no significance in any group. *P < 0.05 was considered a significant difference compared to the control group (i.e., day 0).

Flow cytometry analyses of annexin V and PI were performed to evaluate MVs effects on fibroblast death (Fig. 3). The routine cell proliferation resulted in increased percentages of live cells in both treated and untreated groups of day 3 and day 5 compared to the control group (i.e., 24 h cell culture with no treatment). There was a significant difference in live cell numbers between day 0 and day 3/MVs-. MVs treatment significantly decreased the late apoptosis on both day 3 and day 5 compared to day 0, *p*-value = 0.036735 and 0.016857, respectively. In MVs + groups, early apoptosis was more than in their related untreated groups. There were no significant differences in early apoptosis and necrosis between untreated and MVs treated groups for both time points compared to day 0. In addition, differences in live, early, and late apoptosis, and necrosis on day 5/MVs- compared to day 3/MVs- and day 5/MVs + compared to day 3/MVs + were not significant (Fig. 3C).

The MTT result was in accordance with the increase in early apoptosis data. The absorption of MVs-treated cells at 570 nm was lower than the blank control (0-day MVs), which might result from a decrease in proliferation, or viability (Fig. 3D). In accordance, Trypan blue results also showed no significant difference between cell numbers in MVs + and MVs- groups; however, the cell number slightly decreased under MVs treatment (Fig. 3E).

### The effects of MVs on the transcription of ECM proteins

**Figure 4:**
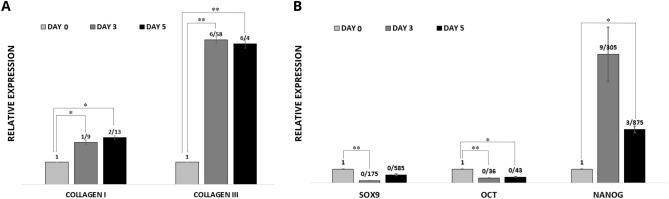
Evaluating transcriptional changes of some stemness markers in MVs treated cells. (**A**) Skin ECM marker genes (collagen I and III) mRNA expression in treated fibroblasts; Both markers were significantly upregulated under MVs treatment. (**B**) Stemness gene markers (Sox9, Oct4, and Nanog) mRNA expression in treated MSCs; Results suggested that MVs down-regulated Sox9 and Oct4, and up-regulated Nanog transcription. Data are presented as means ± SD of at least three independent experiments. *P < 0.05 and **P < 0.01 was considered significant differences compared to the control.

Qualitative PCR results showed that hiPSCs-derived MVs treatment significantly increased collagen Iα1 and collagen III mRNAs expression in fibroblasts on days 3 and 5 of culture (Fig. 4A). Noteworthy collagen III was transcribed nearly three times more than collagen Iα1 in each time point.

### The effects of MVs on stemness properties of MSCs

To evaluate the effects of MVs on stem cells, the stemness properties of MVs treated MSCs were identified by analysis of Sox9, Oct4, and Nanog mRNAs on days 0, 3, and 5 of culture. As presented in Fig. 4B, the patterns of transcriptional changes in the abovementioned markers were different. While Nanog expression increased significantly on day 3 with the following decrease to day 5, Sox9 showed the opposite pattern. Oct4 transcription represented a significant and continued to decrease during treatment.

### The effects of MVs treatment on wound appearance

**Figure 5:**
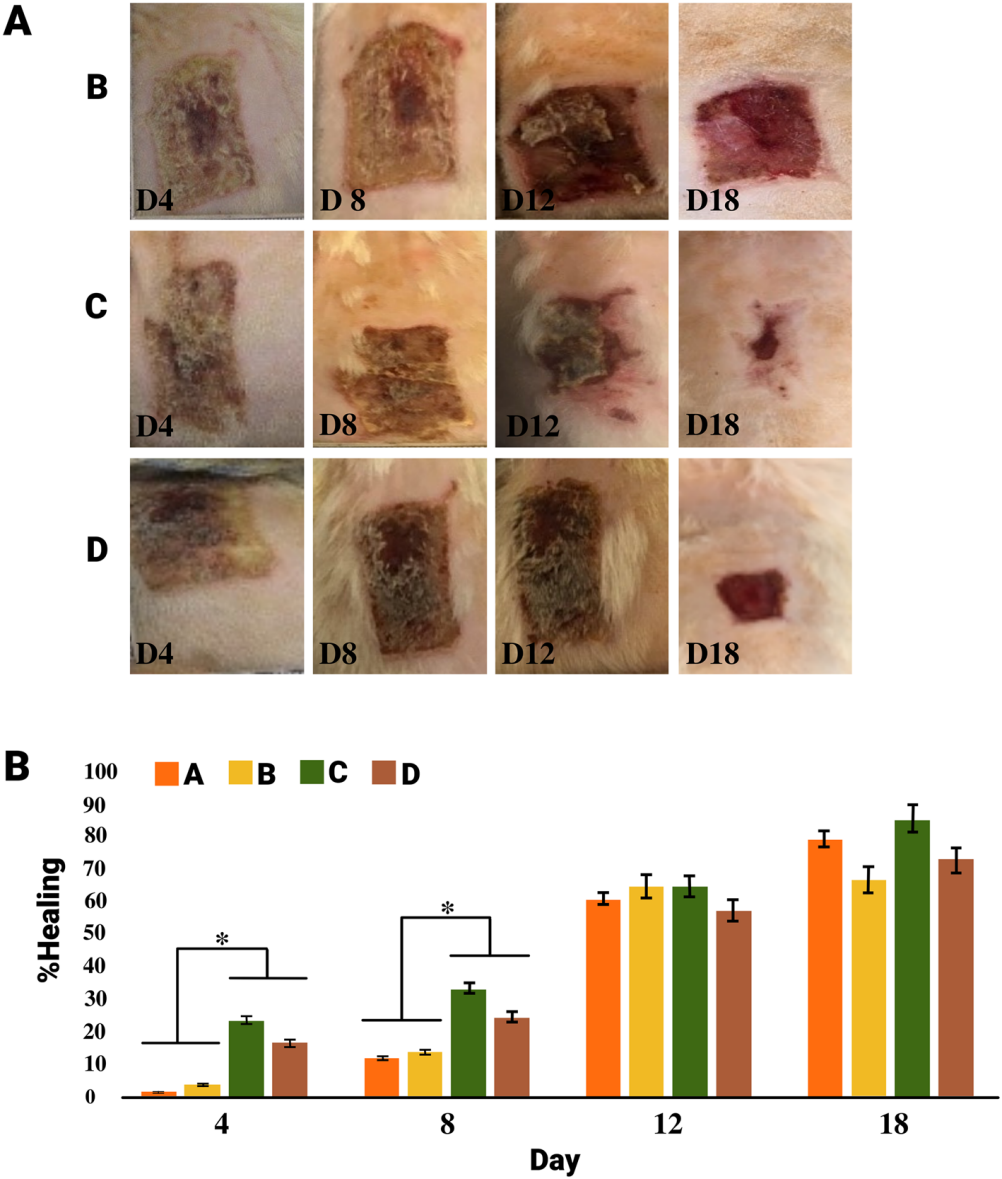
(**A**) Photographs were taken from rat skins in different groups and days to evaluate the changes in wound-healing. Group B: a wound created on the dorsal skin with pure Eucerin lotion (untreated group), Group C: a wound created on the dorsal skin with 0.5% MVs + Eucerin lotion treatment, and Group D: a wound created on the dorsal skin with 1% MVs + Eucerin lotion treatment. (**B**) The diagram represents the percentages of wound-healing in the study groups over a period of 18 days, *P < 0.05.

The size of the wound area in the experimental groups during 18-days was calculated using software AxioVision Rel 4.8.2 (Fig. 5). According to the analysis of images, the significant effects of MVs treatment was particularly detected during the initial stages of wound-healing (days 4 and 8).

### The histological effects of MVs on re-epithelialization and blood vessel formation

**Figure 6:**
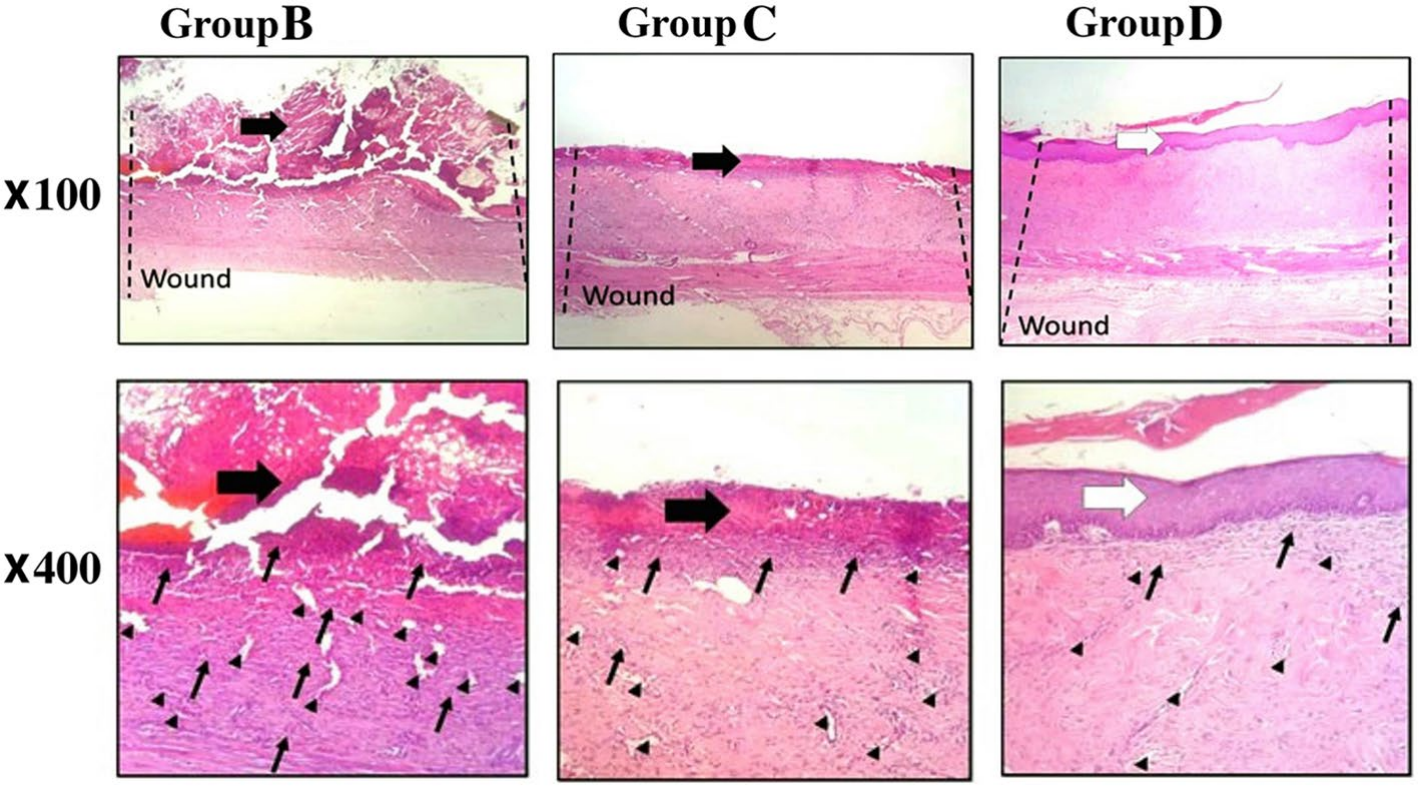
H&E-stained microscopic sections of healed incisions on day 18 of treatments in different groups. Thin arrows: infiltration of inflammatory cells, black thick arrows: crusty scab, white arrows: epithelial layer, Arrowhead: neo-vascularization. Group A as control group is not shown. Group B: a wound created on the dorsal skin with pure Eucerin lotion (untreated group), Group C: a wound created on the dorsal skin with 0.5% MVs + Eucerin lotion treatment, and Group D: a wound created on the dorsal skin with 1% MVs + Eucerin lotion treatment, scale bars are drawn on the images.

Histological analysis of burn injuries showed polymorphonuclear inflammatory cells infiltration, granulation, tissue formation, and the undeveloped epidermis layer with a wound covered by a crusty scab in group B (negative control), in which the rats received Eucerin treatment with no MVs (Fig. 6). The results of the histopathological analysis in group C (treated by 0.5% MVs + Eucerin) indicated a high similarity to results of group B. Similarly, inflammation and visible crusty scab were observed in the wound area without any epidermis formation. A remarkable decrease in the inflammatory cells was detected in group C compared to the control group (group A) at the same time point.

Histopathology of burn wounds in group D (treated by 1% MVs + Eucerin) indicated epidermal proliferation. An increase in the epidermal layer was also detected at the end of the experiment. In addition, a decrease in the inflammatory response and granulation tissue was observed in this group. Compared to the control and other experimental groups, more similar healing to the normal skin tissue was obtained in group D, although in which the epidermal layer was thicker than the normal skin.

According to the results of the histomorphometry analysis of the skin injuries (Table 2) during 18-days treatment, the minimum re-epithelialization was observed in group B mostly which was filled with immature granulation tissue (*P* < *0.05*). The best re-epithelialization was found in group D among the other groups. The number of blood vessels was significantly reduced in group D in comparison to others, which means the higher remodeling of granulation tissue (*P* < *0.001*).

**Table 2:** Epiltheliogenesis scores and the number of blood vessels in different experimental groups.

Group	Epitheliogenesis score	Blood vessels	Inflammatory cells
B	0,1,1,0	24.7 ± 5.0	142.7 ± 13.1
C	1,0,1,1	16.2 ± 2.8*	88.0 ± 13.5**
D	3,4,2,3**	7.7 ± 2.5**	20.2 ± 4.8***

*, **, ***: values indicate treatment group versus negative control groups (empty control); * P < 0.05, ** P < 0.01, *** P < 0.001.

## Discussion and future remarks

Due to the probable disadvantages of allogeneic skin grafting in terms of graft rejection and infection transmission, autologous cell transplantation has become a favorable strategy for wound-healing^39^. In skin injuries, stem cells are essential in re-epithelialization and repair. Also stem cells reduce the inflammation in the burned site, and accelerate the wound-healing by angiogenesis^40^. Currently, application of stem cell-based therapies in the clinical skin healing has been hindered due to being expensive, low scalability with potential safety concerns^18^.

Using hiPSCs could have some outstanding feasibility and advantages over other stem cells, including not stimulating inflammatory responses, low probability of immune rejection, high metabolic activity, good large-scale production capacity and potentials for personalized medicine^17^. There are numerous reports about the impact of MVs on various biological processes since exosomes and MVs can transfer the mentioned molecules across the cells^28,41,42^. Interestingly, hiPSCs secret and shed MVs 16 times more than MSCs^43,44^.

Several studies have reported the influence of iPSCs-derived MVs on transmission of cytoprotective signals to cells^34^. It has been shown that hiPSCs-derived MVs were able to deliver cardioprotective miRNAs to avoid apoptosis of the cardiomyocytes in the ischemic myocardium^44^. Scientists have also reported that hiPSCs-derived MVs could exert protective and proliferative effects on cardiac mesenchymal stromal cells by transferring RNAs and proteins to injured tissues^45^. In another study conducted by Liu et al., hiPSCs-derived MVs caused a decrease in reactive oxygen species by delivering intracellular peroxiredoxin antioxidant enzymes to human senescent MSCs, which subsequently resulted in the alleviation of cellular aging in culture^43^.

Hence, this study aimed to evaluate the possibility, safety and effectiveness of applying iPSCs-derived MVs for skin tissue engineering and rejuvenation applications. The possibility was assessed using the evaluation of the mRNA content of iPSC-derived MVs and the behavior of fibroblasts after MV treatment. Investigating the effect of microvesicle on stemness potential of mesenchymal stem cells was performed for safety concerns. In vivo evaluation of MVs was done in order to investigate related immune response, re-epithelialization and blood vessel formation to measure effectiveness (Fig. 1).

Similar to previous studies^43,46^, isolated hiPSCs-derived MVs size ranges by DLS showed 100–1000 nm in diameter. Also, TEM image of a single isolated MVs demonstrated rounded shape (Fig. 2).

To support the hypothesis that hiPSCs-derived MVs contain wound-healing messengers, first we investigated some important mRNAs inside the hiPSCs-derived MVs, including AQP3, COL2A, FGF2, ITGB, and SEPTIN4, that involve in wound-healing mechanisms seriously^8–14^. AQP3 is a transporter that is highly expressed in the plasma membrane of skin epidermal cells. AQP3, as a water channel, facilitates cell migration. Also, it increases keratinocyte promotion and differentiation through its role as a glycerol receptor. The mentioned functions help AQP3 affect the wound-healing process^47^. COL2A1 contains the pro-alpha1(II) chain that makes this ECM protein a supportive and strengthening component of connective tissues and skin^48^. FGF2 is involved in mitogenesis and angiogenesis, besides recruiting and homing stem cells in injured parts of the body^49^. ITGB, as a member of the integrins family, serves as extracellular matrix receptors and regulate both inside-out and outside-in signaling. Outside-in signaling adjusts keratinocyte responses to microenvironmental changes while inside-out signaling regulates keratinocyte-mediated changes to the wound microenvironment. Integrins, as mediators of cell adhesion and migration, control cell proliferation, survival, and matrix remodeling, along with paracrine crosstalk with other cellular compartments in wound-healing^50^. Sept4 plays a critical role in the regulation of hair follicle stem cells, which results in significant consequences for wound-healing and skin regeneration^51^. Our data confirmed the existence of the mentioned transcripts in hiPSCs-derived MVs, which indirectly supports the possibility of applying MVs for wound-healing. Noteworthy, the isolated MVs may have both direct (i.e., the transmission of growth factors and transcripts) and indirect (i.e., stem cell recruitment) effects on skin regeneration.

Some stem cells are presented in the skin and any damage to their stemness capacity would hinder the skin regeneration. Therefore, we aimed to investigated the possible effect of MVs on stemness potential of stem cell for safety concerns. Based on our experiment, hiPSCs-derived MVs caused no significant alteration in transcriptions of Sox9 and Oct4 during the 5-day treatment (Fig. 4). Notable, MVs treatment upregulated Nanog transcription in stem cells (Fig. 4). Nanog, Sox9, and Oct4 are the core network of transcription factors supporting the stemness state; increased expression of Nanog correlates with high self-renewal efficiency, and the influence of Nanog on the promoter activity of Sox9 and Oct4 is significantly minimal compared to the Sox9/Oct4 influence on Nanog expression^52–54^. Therefore, it could be concluded that MVs were unlikely to have an adverse significant effect on the stemness potential of stem cells.

Fibroblasts have a crucial role in wound healing. They produce matrix metalloproteinases and plasmin which are increased in remodeling of an injured area after a wound^55^. Fibroblasts are considered the highly frequent cells in animal connective tissues, such as in the skin dermis; so, we chose this cell to evaluate our hypothesis effectiveness^56,57^. According to Trypan blue and MTT assays, MV treatments had no significant negative impacts on cell viability and proliferation or metabolic activity rates. Interestingly MV treatment significantly decreased the late apoptosis in the cells (Fig. 3). To sum up, MV could help fibroblast survival and maintenance.

Aging leads to skin collagen decrease and deformation, and subsequent wrinkle formation^58,59^. Collagens are among the major structural components of the skin, and fibrillary collagens (type I and III) that mediate mechanical tension of the skin are abundant in the skin ECM^60–62^. Type I collagen is the most protein in the dermis produced by fibroblasts, synthesizing other collagens (III, V, VII), elastin, proteoglycans, and fibronectin too. The half-life of type I collagen in human skin was shown to be greater than 1 year. In our study, MVs treatment resulted in significant upregulation in the transcription of collagen Iα1 and collagen III in fibroblasts (Fig. 4); which strengthens the encouraging role of MVs in skin rejuvenation.

In clinics, modulated inflammatory response, decreased release of cytokines, and reduced granulation tissue are the hallmarks of a successful cure for skin repair. Antimicrobial defense is required in skin wound-healing; however, the extra inflammatory reactions can cause increased fibrotic response^63,64^. A study showed that applying adipose stem cell-derived MVs caused improvement and promotion of the wound-healing process through angiogenesis and re-epithelization in animal wound models^65^. Accordingly, we investigated the role of hiPSCs-derived MVs in wound-healing in vivo. In our study, MVs treated groups (i.e., groups C and D) compared to control groups (i.e., groups A and B) showed less inflammatory response and granulation along with more epidermalization and skin angiogenesis. By histopathological and histomorphometry analyses, group D (treatment by 1% MVs + Eucerin) revealed a detectable increase in the epidermal layer and a decline in both inflammatory response and granulation tissue. Furthermore, there was inflammation in the wound area of group C (treatment by 0.5% MVs + Eucerin), however, it was lower in comparison with group A. There was no epidermal layer observed in both groups B and C, and granulation tissue was also generated and inflammatory cells were observed in both groups. Overall, in line with in vitro results, histomorphometry and histopathology findings in the rat burn wound models also confirmed the helpful effect of MVs in skin regeneration (Figs. 5 and 6, Table 2).

Taken together, hiPSCs-derived MVs might be a potent treatment for skin tissue engineering and rejuvenation due to confirmed possibility, safety and effectiveness. For the first time, it has been demonstrated that the above-mentioned MVs could cause upregulation of collagen Iα1 and collagen III mRNA expression while having no adverse impacts on the survival and proliferation of both MSCs and fibroblasts. In vivo findings further confirmed the significant effect of MVs treatment in the wound-healing process. These observations have turned hiPSCs-derived MVs into a suitable candidate for skin regeneration.

Identifying the components of iPSCs-derived MV and their effects on the viability, proliferation and expression of ECM markers give some benefits for biomimetics. In this regard, reverse engineering can be applied to produce synthetic defined MVs with clear, safe, and identified components, for skin regeneration approaches. As a prospect to achieve permissions for clinics, more extensive researches such as investigation of the effects of MVs on other skin cells (such as keratinocytes), identification of the whole internal and surface components of MVs (such as miRNAs, other mRNAs, and proteins), and preclinical evaluations of reverse-engineered MVs on skin regeneration are promising.

## Supplementary Information

Supplementary Tables.

## Data Availability

All data generated or analyzed during this study are included in this published article.

## Abbreviations

GAPDH: Glyceraldehyde 3-phosphate dehydrogenase
AQP3: Aquaporin-3
COL2A: Collagen, type II, alpha 1
FGF2: Fibroblast growth factor 2
FGF7: Fibroblast growth factor 7
FGFR2: Fibroblast growth factor receptor 2
ITGB1: Integrin beta 1
PPARD: Peroxisome proliferator activated receptor delta
SEPT: Septin
STAT3: Signal transducer and activator of transcription 3
HPRT: Hypoxanthine-guanine phosphoribosyl transferase
Nanog: Homeobox protein NANOG
Oct 4: Octamer-binding transcription factor 4
Sox 9: SRY-related HMG-box 9
iPSCs: Induced pluripotent stem cells
MVs: Microvesicles
MSC: Mesenchymal stem cells
ECM: Extracellular matrix
TEM: Transmission Electron Microscopy
DLS: Dynamic Light Scattering
H&E: Hematoxylin and eosin
FBS: Fetal bovine serum
miRNAs: MicroRNAs
MTT: 3-4,5-Dimethylthiazol-2-yl
