# Supplementary material for: Transcriptomic and in vivo approaches introduced human iPSC-derived microvesicles for skin rejuvenation
Source: Sci Rep. 2023 Jun 20;13:9963. doi: 10.1038/s41598-023-36162-9 (PMC10282097; doi:10.1038/s41598-023-36162-9)
Supplement: Supplementary file 1 — Supplementary Tables. [file 41598_2023_36162_MOESM1_ESM.docx]

Supplementary Table 1. The primers and the size of PCR products.

| Gene | Access No. | Sequences | Amplicon Size (Bp) |
| --- | --- | --- | --- |
| GAPDH | NM_001357943.2 NM_001256799.3  NM_001289745.3 NM_001289746.2  NM_002046.7 | F 5'- TGGTATCGTGGAAGGACTCATG -3'  R 5'- AGTAGAGGCAGGGATGATGTTC -3' | 130 |
| AQP3 | NM_001318144.2  NM_004925.5 | F 5'-TGACCTTTGCCATGTGCTTCC-3'  R 5'-CGAAGTGCCAGATTGCATCAT-3' | 135 |
| Col2A | NM_033150.3  NM_001844.5 | F 5'-CAGGACCAAAGGGACAGAAAGG-3'  R 5'-GGTTCTCCATCTCTGCCACGAG-3' | 166 |
| FGF2 | NM_001361665.2  NM_002006.5 | F 5'-GACCCTCACATCAAGCTACAAC-3'  R 5'-GAAGCCAGTAATCTTCCATCTTCC-3' | 113 |
| FGF7 | NM_002009.4 | F 5'-GTCACAGCAACTGAACTTACTACG-3'  R 5'-TGGAGTCATGTCATTGCAAGC-3' | 358 |
| FGFR2 | NM_000141.5  NM_001144918.2  NM_001144917.2  NM_001320658.2  NM_001144919.2 | F 5'-GTTTAAGCAGGAGCATCGCATTG-3'  R 5'-CTCCACAACATCCAGGTGGTAC-3' | 160 |
| INTEGRIN (ITGB1) | NM_002211.4  NM_133376.2  NM_033668.2 | F 5'-GTGGGTGGTGCACAAATTCAAC-3'  R 5'-ATCGCAAAACCAACTGCTGTGG-3' | 234 |
| PPARD | NM_177435.3  NM_001171818.2  NM_006238.5 | F 5'-TTCTCCAAGCACATCTACAATGC-3'  R 5'-CATTCACCAACTGCTTCCACAC-3' | 169 |
| SEPT (SEPTIN4) | NM_001368771.2  NM_001368772.1  NM_001363803.1 | F 5'-AGTCCACCGAAAGTCCGTGAAG-3'  R 5'-CTCTTCAGCACCAAGAAGTTTCC-3' | 142 |
| STAT3 | NM_001369520.1  NM_001369519.1  NM_001369518.1  NM_001369517.1  NM_001369516.1  NM_001369514.1  NM_001369513.1  NM_001369512.1 | F 5'-CTTCTACAGACTGCAGCCACTG-3'  R 5'-TTCTAGATCCTGCACTCTCTTCC-3' | 135 |
| HPRT | NM_013556.2 | F 5'-TCCCAGCGTCGTGATTA-3'  R 5'-CGAGCAAGTCTTTCAGTC-3' | 138 |
| NANOG | NM_028016.3  NM_001289828.1 | F 5'-TGATTTGGTTGGTGTCTT-3'  R 5'-TGTGATGGCGAGGGAA-3' | 176 |
| SOX9 | NM_011448.4 | F 5'-CAGCAAGAACAAGCCACA-3'  R 5'-GTCTCTTCTCGCTCTCGTT-3' | 164 |
| OCT4 | NM_001252452.1  NM_013633.3 | F 5'-GTTCTCTTTGGAAAGGTGTT-3'  R 5'-GCATATCTCCTGAAGGTTCT-3' | 147 |
| Collagen I | NM_033150.3  NM_001844.5 | F 5'- GAGCCAAAGGATCTGCTGGT -3'  R 5'-TTGGGGCCTTGTTCACCTTT-3' | 160 |
| Collagen II | NM_000090.4 | F 5'-TATCGAACACGCAAGGCTGT -3'  R 5'-AAAAGCAAACAGGGCCAACG-3' | 106 |
